# Sex-specific structural and functional cardiac remodeling during healthy aging assessed by cardiovascular magnetic resonance

**DOI:** 10.1007/s00392-024-02430-5

**Published:** 2024-03-11

**Authors:** Leonhard Grassow, Jan Gröschel, Hadil Saad, Leo Dyke Krüger, Johanna Kuhnt, Maximilian Müller, Thomas Hadler, Edyta Blaszczyk, Jeanette Schulz-Menger

**Affiliations:** 1 Charité – Universitätsmedizin Berlin, corporate member of Freie Universität Berlin and Humboldt-Universität zu Berlin, ECRC Experimental and Clinical Research Center, Lindenberger Weg 80, 13125 Berlin, Germany; 2 Working Group on Cardiovascular Magnetic Resonance, Experimental and Clinical Research Center, a joint cooperation between Charité Medical Faculty and the Max-Delbrück Center for Molecular Medicine, Charité Campus Buch, Lindenberger Weg 80, 13125 Berlin, Germany; 3 DZHK (German Centre for Cardiovascular Research), partner site Berlin, Germany; 4 Deutsches Herzzentrum der Charité – Department of Cardiology, Angiology and Intensive Care Medicine, Charitéplatz 1, 10117 Berlin, Germany; 5 HELIOS Hospital Berlin-Buch – Department of Cardiology and Nephrology, Schwanebecker Chaussee 50, 13125 Berlin, Germany

**Keywords:** Age, Women, Sex, Cardiac physiology, Cardiac risk factor, Cardiovascular magnetic resonance

## Abstract

**Background:**

Aging as a major non-modifiable cardiac risk factor challenges future cardiovascular medicine and economic demands, which requires further assessments addressing physiological age-associated cardiac changes.

**Objectives:**

Using cardiovascular magnetic resonance (CMR), this study aims to characterize sex-specific ventricular adaptations during healthy aging.

**Methods:**

The population included healthy volunteers who underwent CMR at 1.5 or 3 Tesla scanners applying cine-imaging with a short-axis coverage of the left (LV) and right (RV) ventricle. The cohort was divided by sex (female and male) and age (subgroups in years): 1 (19–29), 2 (30–39), 3 (40–49), and 4 (≥50). Cardiac adaptations were quantitatively assessed by CMR indices.

**Results:**

After the exclusion of missing or poor-quality CMR datasets or diagnosed disease, 140 of 203 volunteers were part of the final analysis. Women generally had smaller ventricular dimensions and LV mass, but higher biventricular systolic function. There was a significant age-associated decrease in ventricular dimensions as well as a significant increase in LV mass-to-volume ratio (LV-MVR, concentricity) in both sexes (LV-MVR in g/ml: age group 1 vs. 4: females 0.50 vs. 0.57, *p*=0.016, males 0.56 vs. 0.67, *p*=0.024). LV stroke volume index decreased significantly with age in both sexes, but stronger for men than for women (in ml/m^2^: age group 1 vs. 4: females 51.76 vs. 41.94, *p*<0.001, males 55.31 vs. 40.78, *p*<0.001). Ventricular proportions (RV-to-LV-volume ratio) were constant between the age groups in both sexes.

**Conclusions:**

In both sexes, healthy aging was associated with an increase in concentricity and a decline in ventricular dimensions. Furthermore, relevant age-related sex differences in systolic LV performance were observed.

**Graphical Abstract:**

↓, decrease; ↑, increase; ±, maintaining. Abbreviations: CMR, cardiovascular magnetic resonance; EDV, end-diastolic volume; EF, ejection fraction; LV, left ventricle; MVR, mass-to-volume ratio; RV, right ventricle; SVI, stroke volume index; T, Tesla; VR, volume ratio.

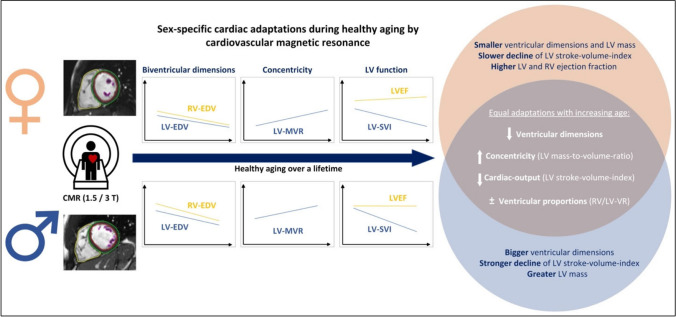

**Supplementary Information:**

The online version contains supplementary material available at 10.1007/s00392-024-02430-5.

## Introduction

The prevalence of cardiovascular diseases increases with rising age [1, 2]. Global demographic change will likely impact not just patient’s health but also lead to an increasing burden on healthcare resources [2]. Therefore, research into the physiological role of non-modifiable risk factors such as age and sex becomes increasingly important. It is known that left ventricular (LV) remodeling occurs in patients with underlying cardiac diseases such as myocardial infarction [3]. Data also suggest that rising age is associated with LV remodeling [4, 5] including an increased occurrence of LV fibrosis [6], stiffness [7], and wall thickening [8, 9], turning age into one of the strongest cardiovascular risk factors for the development of cardiovascular diseases and particularly heart failure (HF). Sex-specific differences in the general and age-associated cardiac morphology are also reported in literature: Female hearts tend to be more affected by concentric than eccentric remodeling [10, 11], by a greater increase in LV wall thickening [7, 11, 12] and diastolic dysfunction [11, 13, 14] compared to male hearts, which is also reflected in the clinical phenotype of HF. Women display a higher prevalence of HF with preserved ejection fraction, whereas men are more likely to develop HF with reduced ejection fraction [15].

Nevertheless, data regarding the age-related progression of LV systolic performance and LV mass (LVM) are discordant and also vary in their methodology [8, 9, 16, 17]. Potential age-related cardiac changes should be investigated across all life stages and with equal distribution of sex, particularly as women are underrepresented in cardiovascular research [18] and appear to show a higher in-hospital mortality rate (as assessed in a cohort with myocardial infarction) [19]. Further investigation of the impact of sex and age on cardiac geometry and function will improve the understanding of physiological ventricular adjustments over the adult lifetime and could provide possible explanations for sex differences in cardiac morbidity and mortality.

Since cardiovascular magnetic resonance (CMR) has been established as the gold standard for the quantification of cardiac chambers as well as for myocardial tissue characterization [20], it is highly qualified to evaluate cardiac changes during healthy aging. Quantitative CMR parameters provide the basis for ventricular and atrial assessment and allow the differentiation of pathological from physiological conditions. Indexed and related performance parameters, such as the LV mass-to-volume ratio (LV-MVR) as a predictor of concentricity, the LV stroke volume index (LV-SVI) as a determinant of cardiac output, and the ratio of right ventricular (RV) to LV end-diastolic volumes (RV/LV-VR) to evaluate the proportion of ventricular dimensions, can provide adequate diagnostic as well as prognostic [21–23] information.

This study aims to characterize physiological age-dependent cardiac adaptations in ventricular geometry and function between healthy women and men by using quantitative volumetric and functional indices in CMR.

## Methods

### Study design

This research was designed as a retrospective analysis of CMR image data in a cohort of consecutively included healthy volunteers. All data were collected in previous prospective clinical trials between 2011 and 2023. These studies were approved by the ethics committee of the Charité University Medicine Berlin, Germany, and were subject to the ethical standards of the institution and the Declaration of Helsinki.

### Study population

The study cohort included adult Caucasians who were enrolled as healthy controls in previous prospective studies. Participants who displayed or developed cardiovascular symptoms or diseases were consequently excluded from the cohort using a standard operating procedure (supplementary methods 1). Exclusion criteria were further extended to conditions beyond the cardiovascular system and post-analysis detected abnormalities. Thus, the final analysis covered individuals who exhibited entirely healthy conditions. Detailed information regarding the sample enrollment and the specific conditions leading to an exclusion are provided in supplementary methods 1. The total cohort was divided into different age groups (in years: 1, 19–29; 2, 30–39; 3, 40–49; 4, ≥50; adjusted after Kawel-Boehm et al. [24]) and divided by biological sex (female, male).

### Image acquisition

CMR examinations were performed at 1.5 Tesla (Avanto and AvantoFit, Siemens Healthineers, Erlangen, Germany) and 3 Tesla scanners (SkyraFit and Verio, Siemens Healthineers, Erlangen, Germany). Following general anatomical overviews and localizers, the CMR protocol included ECG-gated cine imaging with a balanced steady-state free precession (bSSFP) sequence in a short-axis stack covering the entire LV and RV. Technical CMR sequence details are given in supplementary methods 2.

### Image analysis

The CMR image analysis was performed using the dedicated post-processing software CVI42 (Circle CVI, version 5.13.7, Calgary, Canada). The evaluation proceeded in two phases: First, pre-existing manually generated or missing LV and RV contours from the initial studies were reworked and updated by an experienced CMR reader following a standard approach according to current scientific recommendations [20]. In a second supervision, all images were checked by two qualified CMR experts.

For short-axis biventricular assessment, endo- and epicardial borders were contoured in end-diastolic and end-systolic phases. Basal slices were counted as part of the LV blood volume if they were surrounded by at least 50% of the myocardium. LV papillary muscles were contoured in end-diastolic and end-systolic phases and counted as part of LVM.

**Fig. 1:**
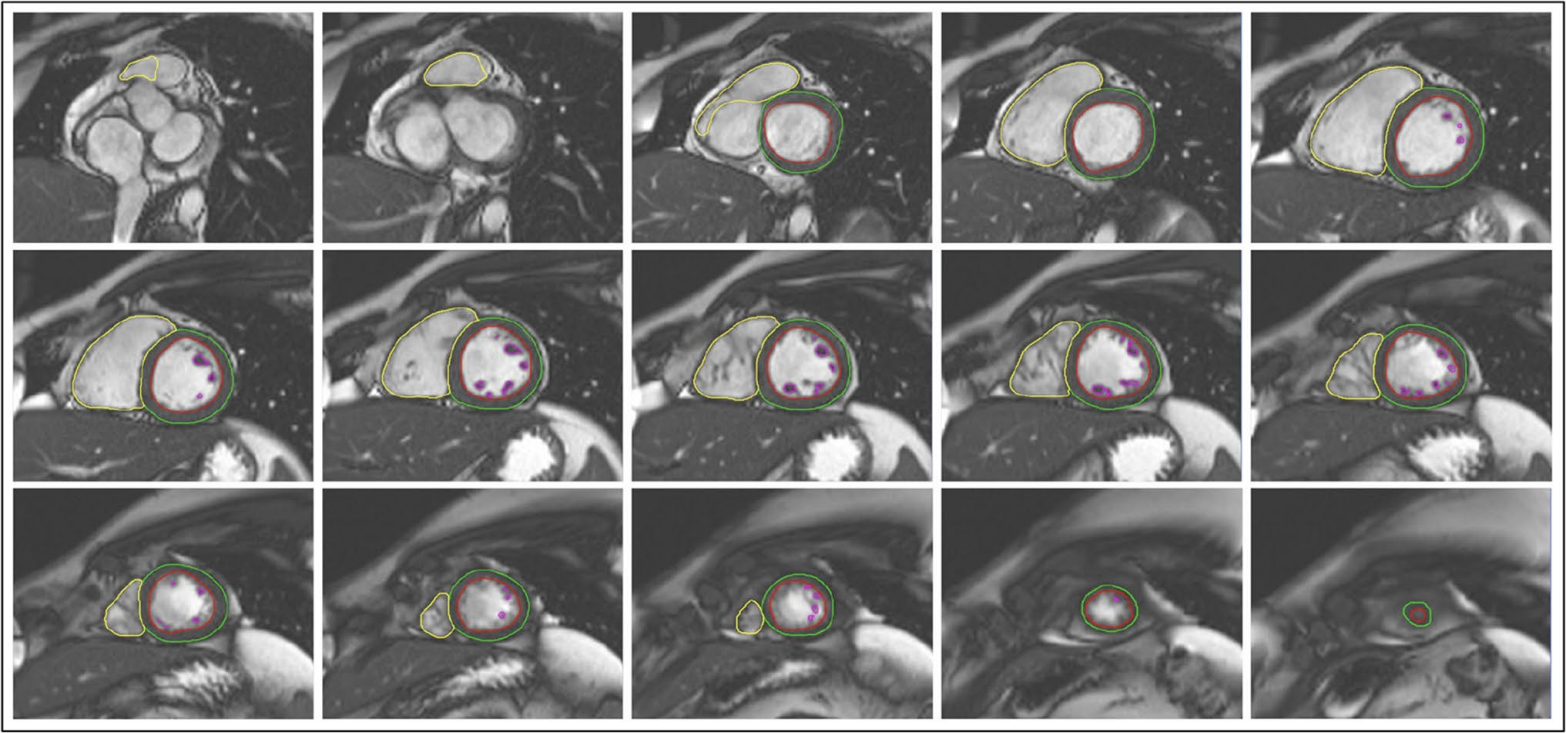
Exemplary illustration of a complete cine short-axis stack contouring in LV and RV end-diastolic phases. Yellow contour: RV endocardial contour, red contour: LV endocardial contour, green contour: LV epicardial contour, purple contour: LV papillary muscle contour. Abbreviations: LV, left ventricle; RV, right ventricle

A general visual assessment of image quality was integrated into the image analysis. Referring to the image quality criteria by Klinke et al. [25], scans that displayed major artifacts (e.g. image blurring, mis-triggering, or wrap-around artifacts) were excluded from the cohort. Figure 1 illustrates a full short-axis stack contouring in the LV and RV end-diastolic phase.

### Quantitative CMR performance parameters

This study assessed ventricular adaptations during aging with a focus on indexed and related CMR parameters such as the LV-SVI, the LV-MVR, and the RV/LV-VR. LV-MVR was determined by the division of LVM and LV end-diastolic volume and RV/LV-VR by the division of RV end-diastolic volume and LV end-diastolic volume. Other parameters were indexed to both height (H) (m) and body surface area (BSA) (m^2^).

### Statistical analysis

The statistical calculations were carried out in the software SPSS (IBM, version 28.0.0, Armonk, NY, USA). Continuous parameters were represented as mean ± standard deviation (SD), categorical variables as total, and percent (%). Normal distributions were assessed with the Shapiro-Wilk test. For the age-independent sex comparison, pairwise tests were performed using the Mann-Whitney *U* test for non-parametric variables and an independent sample *t*-test for parametric variables. The overall age group comparison was carried out by the Kruskal-Wallis test for non-parametric and by ANOVA for parametric parameters. For cases in which significance was detected, an adjusted pairwise post-hoc test followed the assessment. Categorial variables were compared using the chi-square test. Age-correlation analysis was carried out by Spearman’s rank correlation coefficient. A *p*-value ≤0.05 was defined as statistically significant.

### Intra- and interobserver variability

For analyzing intra- and interobserver variability, the in-house developed software tool “Lazy Luna” was used, which enables automatic comparison of CMR segmentations between different readers [26, 27]. Mean deviation ± SD and Bland-Altman plots were calculated based on a subgroup of ten randomly selected subjects for biventricular volumetric and functional parameters.

## Results

### Baseline characteristics

A total of 203 volunteers were screened for this study. Before performing the image analysis, *N*=56 had to be excluded due to cardiovascular symptoms, diseases, conditions affecting other body systems, or missing CMR image data. After image analysis, another *N*=7 subjects were excluded due to diseases detected during post-analysis or artifacts leading to the inability of analysis. The final cohort covered *N*=140 healthy volunteers (Supplementary methods 1).

The cohort presented the following baseline characteristics (Table 1): Age ranged from 19 to 77 years with a cohort’s middle age ± SD of 37.73 ± 13.71 years. Females: *N*=77 (55%), mean age in years ± SD 39.60 ± 14.31; female age groups (in years ± SD): 1, *N*=20, 24.55 ± 2.96; 2, *N*=25, 33.80 ± 3.12; 3, *N*=13, 43.54 ± 3.55; 4, *N*=19, 60.37 ± 8.32. Males: *N*=63 (45%), mean age in years ± SD 35.44 ± 12.68; male age groups (in years ± SD): 1, *N*=25, 24.68 ± 2.87; 2, *N*=21, 33.57 ± 2.68; 3, *N*=7, 45.71 ± 3.55; 4, *N*=10, 59.10 ± 5.20. Height, weight, body mass index (BMI), and BSA were significantly higher in males (Table 1). Baseline characteristics of the sex-specific age groups are provided in Table 2.

**Table 1:** Baseline characteristics

Parameter	Female	Male	*p*-value
	Mean ± SD/absolute (%)	Mean ± SD/absolute (%)	
*N*	77 (55%)	63 (45%)	
*Age (years)*	39.60 ± 14.31	35.44 ± 12.68	0.061^†^
*Height (cm)*	168.27 ± 5.67	181.56 ± 7.48	**<0.001**^**†**^
*Weight (kg)*	65.57 ± 9.26	80.12 ± 11.82	**<0.001**^**†**^
*BMI (kg/m*^*2*^*)*	23.20 ± 3.46	24.31 ± 3.35	**0.011**^**†**^
*BSA (m*^*2*^*)*	1.75 ± 0.13	2.00 ± 0.17	<0.001*
*RR syst. (mmHg)*	126.86 ± 15.08	126.30 ± 12.13	0.775^†^
*RR dias. (mmHg)*	72.49 ± 10.50	75.54 ± 8.29	0.147*
*Heart rate (bpm)*	70.66 ± 9.09	71.45 ± 12.34	0.676*
*MRI scanner (1.5 T/3 T)*	69 (49.3%) / 8 (5.7%)	49 (35.0%) / 14 (10.0%)	0.056^‡^

*BMI* body mass index, *BSA* body surface area, *MRI* magnetic resonance imaging, *RR dias. *diastolic blood pressure, *RR syst.* systolic blood pressure, *SD* standard deviation, *T* Tesla. Statistics: *t-test, ^†^Mann-Whitney *U* test, ^‡^chi^2^-test. *P*≤0.05 defines statistical significance (bold)

**Table 2:** Baseline characteristics of female and male age groups

Parameter	Age group 1 (19–29 years)	Age group 2 (30–39 years)	Age group 3 (40–49 years)	Age group 4 (≥50 years)	*p*-value
**Female**					
* N*	20	25	13	19	
	**Mean ± SD**	**Mean ± SD**	**Mean ± SD**	**Mean ± SD**	
* Height (cm)*	169.04 ± 6.32	168.80 ± 5.37	166.76 ± 6.52	167.79 ± 4.91	0.658*
* Weight (kg)*	61.23 ± 6.44	67.32 ± 10.97	65.39 ± 6.81	67.95 ± 9.79	0.116^†^
* BMI (kg/m*^*2*^*)*	21.44 ± 2.13	23.67 ± 4.00	23.63 ± 3.26	24.14 ± 3.55	**0.031**^†^: *1–4: 0.043*
* BSA (m*^*2*^*)*	1.69 ± 0.11	1.77 ± 0.14	1.74 ± 0.09	1.77 ± 0.13	0.142^†^
**Male**					
* N*	25	21	7	10	
	**Mean ± SD**	**Mean ± SD**	**Mean ± SD**	**Mean ± SD**	
* Height (cm)*	181.72 ± 9.20	183.76 ± 4.59	182.71 ± 4.61	175.70 ± 6.93	0.039*: *2–4: 0.028*
* Weight (kg)*	73.55 ± 9.46	84.84 ± 13.58	83.60 ± 4.37	84.17 ± 10.08	0.003*: *1–2: 0.005*
* BMI (kg/m*^*2*^*)*	22.26 ± 2.26	25.11 ± 3.93	25.06 ± 1.46	27.20 ± 2.23	<0.001*: *1–2: 0.008, 1–4:<0.001*
* BSA (m*^*2*^*)*	1.92 ± 0.16	2.08 ± 0.17	2.06 ± 0.06	2.01 ± 0.15	0.012*: *1–2: 0.010*

*BMI* body mass index, *BSA* body surface area, *SD* standard deviation. Statistics: *ANOVA with pairwise post-hoc parametric test, ^†^Kruskal-Wallis test with pairwise post-hoc non-parametric test. *P*≤0.05 defines statistical significance (bold)

### Age-independent sex-specific differences in cardiac geometry and function

Males had significantly higher LV and RV volumes overall compared to females. These differences remained after indexing to BSA or H (Table 3).

**Table 3:** Age-independent sex-specific volumetric and functional parameters

Parameter	Female (*N*=77)	Male (*N*=63)	*p*-value
	**Mean ± SD**	**Mean ± SD**	
*LV-EDV (ml)*	134.84 ± 20.72	170.19 ± 33.27	**<0.001**^**†**^
*LV-EDV-I/BSA (ml/m*^*2*^*)*	77.47 ± 12.15	84.99 ± 15.50	0.002*
*LV-EDV-I/H (ml/m)*	80.12 ± 11.89	93.48 ± 16.34	**<0.001**^**†**^
*LV-SV (ml)*	83.59 ± 13.05	102.14 ± 20.79	<0.001*
*LV-SV-I/BSA (ml/m*^*2*^*)*	48.04 ± 7.73	51.00 ± 9.68	0.046*
*LV-SV-I/H (ml/m)*	49.67 ± 7.53	56.12 ± 10.44	**<0.001**^**†**^
*LV-EF (%)*	62.14 ± 4.46	60.04 ± 4.11	0.005*
*LVM (g)*	66.09 ± 10.64	100.73 ± 22.33	**<0.001**^**†**^
*LVM-I/BSA (g/m*^*2*^*)*	37.93 ± 6.02	50.12 ± 9.72	**<0.001**^**†**^
*LVM-I/H (g/m)*	39.27 ± 6.15	55.39 ± 11.67	**<0.001**^**†**^
*LV-MVR (g/ml)*	0.50 ± 0.09	0.60 ± 0.10	<0.001*
*RV-EDV (ml)*	150.09 ± 24.89	195.62 ± 41.24	<0.001*
*RV-EDV-I/BSA (ml/m*^*2*^*)*	86.21 ± 14.38	97.56 ± 18.72	<0.001*
*RV-EDV-I/H (ml/m)*	89.12 ± 14.02	107.42 ± 20.47	<0.001*
*RV-EF (%)*	53.98 ± 4.41	50.82 ± 4.22	<0.001*
*RV/LV-VR*	1.12 ± 0.11	1.15 ± 0.09	**0.036**^**†**^

*BSA* body surface area, *EDV* end-diastolic volume, *EF* ejection fraction, *H* height, *I* index, *LV* left ventricle, *LVM* left ventricular mass, *MVR* mass-to-volume ratio, *RV* right ventricle, *SD* standard deviation, *SV* stroke volume, *VR* volume ratio. Statistics: *t-test, ^†^Mann-Whitney *U* test. *P*≤0.05 defines statistical significance (bold)

Accordingly, LV-SVI (indexed to BSA/H) was significantly higher in males, although LV function as measured by ejection fraction was higher in females (Table 3).

Likewise, LVM was significantly higher in males compared to females. The same was observed after indexing to BSA or H (Table 3). For LV-MVR, males were found to have significantly higher ratios compared to females (Table 3). Furthermore, RV/LV-VR differed significantly between both sexes, but only with a slightly higher mean for males (Table 3). The overall mean for RV/LV-VR was greater than 1.0, indicating larger dimensions of the RV in relation to the LV.

### Age-associated sex-specific adaptations in cardiac geometry and function

**Fig. 2:**
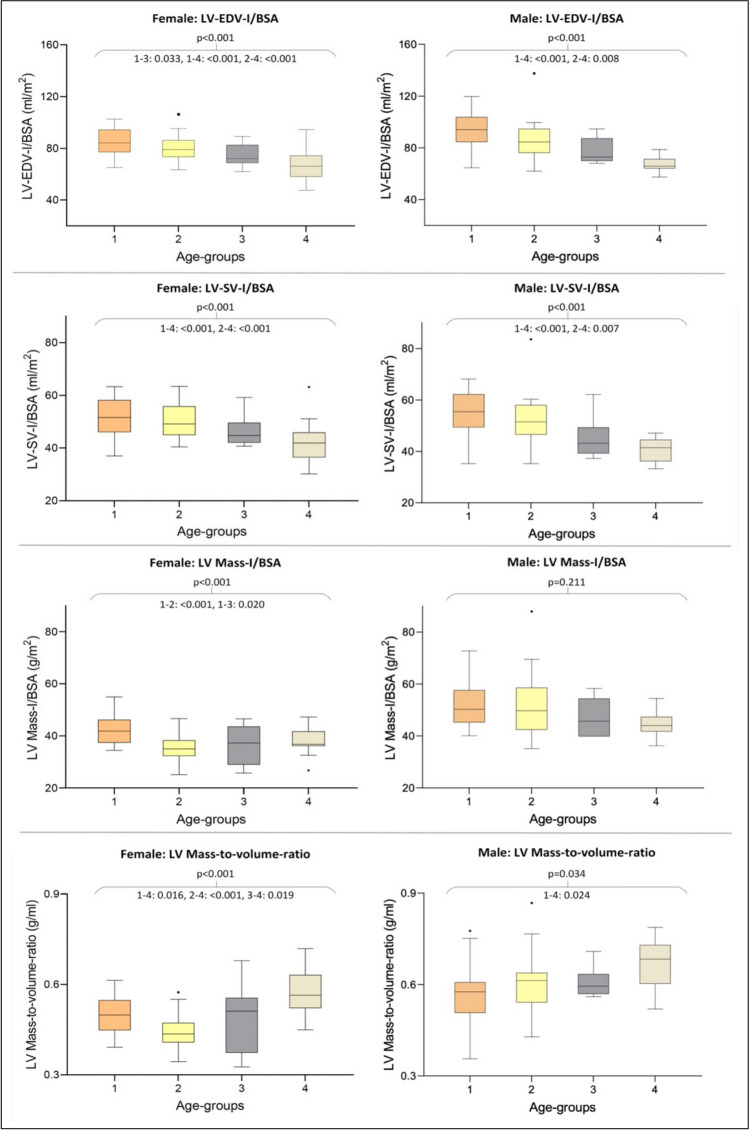
Boxplot comparison of left ventricular CMR indices in female (left) and male (right) age groups. Boxplots represent the median (solid inside the box), interquartile range (box), and 1.5*interquartile range (whiskers). Every value below or above 1.5*interquartile range is marked as an outlier. Age groups in years: 1, 19–29 (orange); 2, 30–39 (yellow); 3, 40–49 (gray); 4, ≥50 (beige). Abbreviations: BSA, body surface area; CMR, cardiovascular magnetic resonance; EDV, end-diastolic volume; I, index; LV, left ventricle; SV, stroke volume

**Fig. 3:**
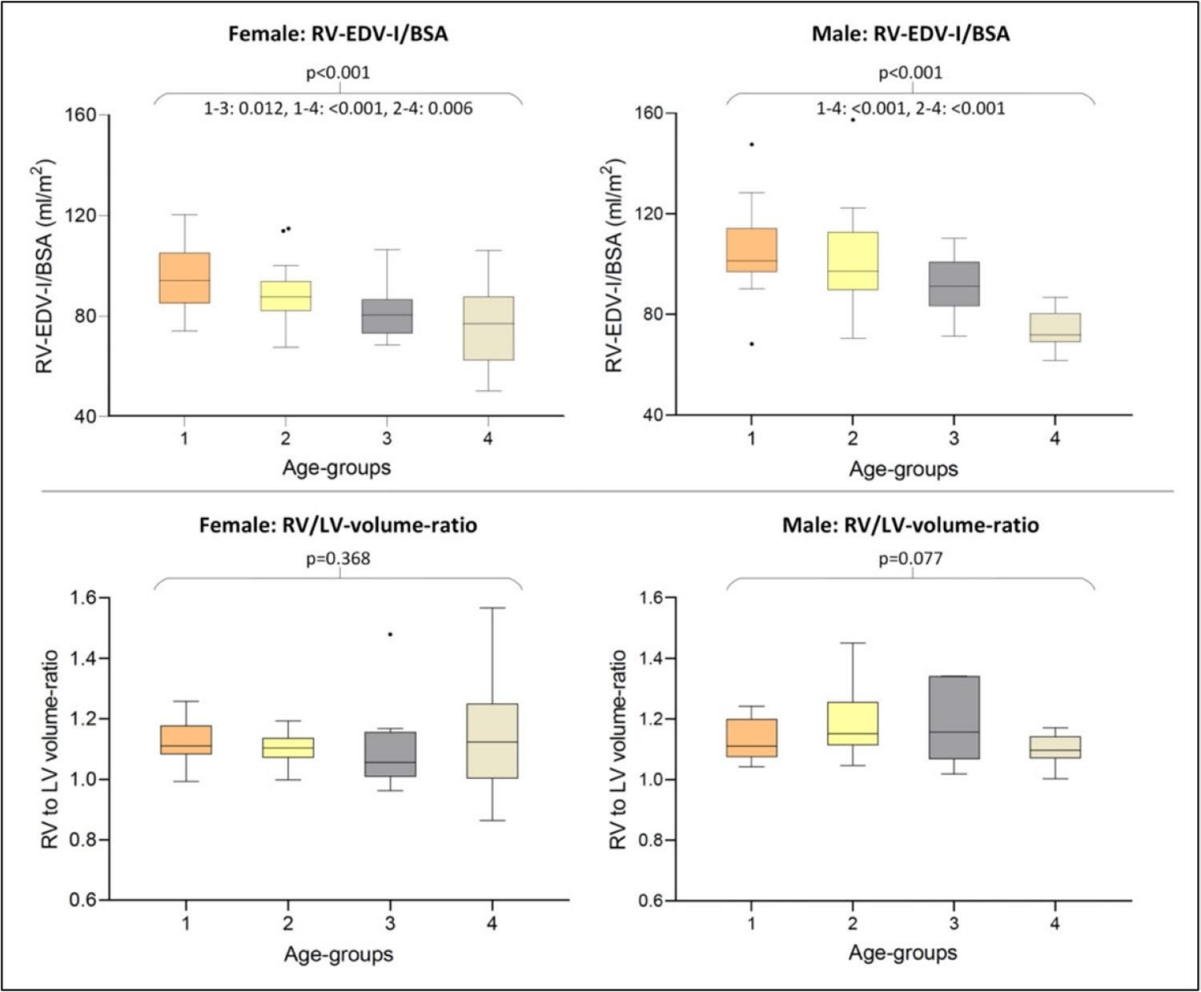
Boxplot comparison of right ventricular CMR indices in female (left) and male (right) age groups. Boxplots represent the median (solid inside the box), interquartile range (box), and 1.5*interquartile range (whiskers). Every value below or above 1.5*interquartile range is marked as an outlier. Age groups in years: 1, 19–29 (orange); 2, 30–39 (yellow); 3, 40–49 (gray); 4, ≥50 (beige). Abbreviations: BSA, body surface area; CMR, cardiovascular magnetic resonance, EDV, end-diastolic volume; I, index; LV, left ventricle; RV, right ventricle

**Fig. 4:**
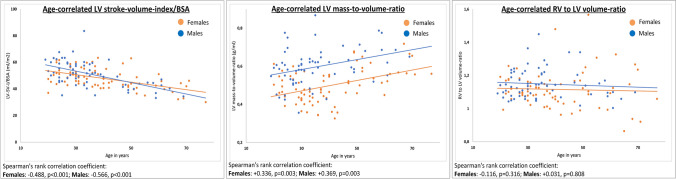
Age-correlated development of LV stroke volume index/BSA, LV mass-to-volume ratio, and RV-to-LV-volume ratio in women and men. Female graphs, orange; male graphs, blue. Abbreviations: BSA, body surface area; I, index; LV, left ventricle; RV, right ventricle; SV, stroke volume

For both sexes, LV and RV volumes and their indexed parameters decreased significantly with increasing age (Tables 4, 5; Figs. 2, 3). The same was evident for LV-SVI (indexed to BSA/H): Younger individuals of both sexes were found to have significantly higher stroke volume indices than older volunteers (Tables 4, 5; Fig. 2). The analysis revealed significant negative age-related correlations for LV-SVI (indexed to BSA/H) for both sexes (Spearman’s rank correlation coefficient: LV-SVI/BSA: females −0.488, *p*<0.001; males −0.566, *p*<0.001; LV-SVI/H: females −0.409, *p*<0.001; males −0.375, *p*=0.002), confirming the decrease of LV-SVI with rising age (Fig. 4, Supplementary results 1). The decreasing LV stroke volume (index) can be explained by a greater age-related decline of LV end-diastolic volume in relation to LV end-systolic volume (Supplementary results 1). The age-correlated LV-SVI/BSA trend curves of females and males crossed at the age of 47.59 years (Fig. 4). Younger males initially presented higher LV-SVI/BSA, but lower mean values than females in advanced life stages (age groups 3 and 4). Therefore, the age-related decline of LV-SVI/BSA was stronger in males (Tables 4, 5; Fig. 4). LV-SVI/H was also found to decrease faster for men than for women with rising age, although the trend curves did not cross (Supplementary results 1).

**Table 4:** Volumetric and functional parameters in female age groups

Female	Age group 1 (19–29 y) (*N*=20)	Age group 2 (30–39 y) (*N*=25)	Age group 3 (40–49 y) (*N*=13)	Age group 4 (≥50 y) (*N*=19)	
**Parameter**	**Mean ± SD**	**Mean ± SD**	**Mean ± SD**	**Mean ± SD**	***p*-value**
* LV-EDV (ml)*	144.40 ± 20.93	142.59 ± 17.05	129.92 ± 13.96	117.92 ± 18.09	<0.001*: *1–4: <0.001, 2–4: <0.001*
* LV-EDV-I/BSA (ml/m*^*2*^*)*	85.24 ± 10.39	80.68 ± 9.48	74.87 ± 8.09	66.85 ± 11.86	<0.001*: *1–3: 0.033, 1–4: <0.001, 2–4: <0.001*
*LV-EDV-I/H (ml/m)*	85.34 ± 11.27	84.42 ± 9.55	77.95 ± 8.33	70.44 ± 11.68	<0.001*: *1–4: <0.001, 2–4: <0.001*
* LV-SV (ml)*	87.54 ± 12.78	89.25 ±11.85	80.76 ± 10.37	73.92 ± 11.02	<0.001*: *1–4: 0.003, 2–4: <0.001*
* LV-SV-I/BSA (ml/m*^*2*^*)*	51.76 ± 7.07	50.49 ± 6.45	46.49 ± 5.66	41.94 ± 7.61	<0.001*: *1–4: <0.001, 2–4: <0.001*
* LV-SV-I/H (ml/m)*	51.77 ± 7.20	52.83 ± 6.44	48.43 ± 6.10	44.16 ± 7.24	**<0.001**^**†**^**:** *1–4: 0.011, 2–4: 0.001*
* LV-EF (%)*	60.77 ± 4.49	62.65 ± 4.43	62.17 ± 4.30	62.89 ± 4.61	0.440*
* LVM (g)*	71.68 ± 11.07	62.40 ± 8.69	63.37 ± 13.32	66.91 ± 8.38	0.020*: *1–2: 0.020*
* LVM-I/BSA (g/m*^*2*^*)*	42.31 ± 5.62	35.31 ± 4.80	36.42 ± 7.21	37.79 ± 4.68	<0.001*: *1–2: <0.001, 1–3: 0.020*
* LVM-I/H (g/m)*	42.37 ± 6.04	36.98 ± 5.18	37.96 ± 7.69	39.91 ± 5.15	0.022*: *1–2: 0.019*
* LV-MVR (g/ml)*	0.50 ± 0.06	0.44 ± 0.06	0.49 ± 0.11	0.57 ± 0.07	<0.001*: *1–4: 0.016, 2–4: <0.001, 3–4: 0.019*
* RV-EDV (ml)*	162.59 ± 25.04	156.85 ± 20.47	141.97 ± 18.51	133.58 ± 24.39	**0.001**^**†**^**:** *1–4: 0.006, 2–4: 0.017*
* RV-EDV-I/BSA (ml/m*^*2*^*)*	95.98 ± 12.65	88.70 ± 11.00	81.77 ± 10.09	75.68 ± 15.15	<0.001^*^: *1–3: 0.012, 1–4: <0.001, 2–4: 0.006*
* RV-EDV-I/H (ml/m)*	96.09 ± 13.69	92.91 ± 11.74	85.03 ± 9.25	79.61 ± 14.47	<0.001*: *1–4: <0.001, 2–4: 0.006*
* RV-EF (%)*	51.94 ± 4.73	55.18 ± 3.94	54.84 ± 3.76	53.97 ± 4.61	0.083*
* RV/LV-VR*	1.13 ± 0.07	1.10 ± 0.05	1.10 ± 0.14	1.14 ± 0.17	0.368^†^

*BSA* body surface area, *EDV* end-diastolic volume, *EF* ejection fraction, *H* height, *I* index, *LV* left ventricle, *LVM* left ventricular mass, *MVR* mass-to-volume ratio, *RV* right ventricle, *SD* standard deviation, *SV* stroke volume, *VR* volume ratio, *y* years. Statistics: *ANOVA with pairwise post-hoc parametric test, ^†^Kruskal-Wallis test with pairwise post-hoc non-parametric test. *P*≤0.05 defines statistical significance (bold)

**Table 5:** Volumetric and functional parameters in male age groups

Male	Age group 1 (19–29 y) (*N*=25)	Age group 2 (30–39 y) (*N*=21)	Age group 3 (40–49 y) (*N*=7)	Age group 4 (≥50 y) (*N*=10)	
**Parameter**	**Mean ± SD**	**Mean ± SD**	**Mean ± SD**	**Mean ± SD**	***p*-value**
* LV-EDV (ml)*	180.25 ± 34.36	177.36 ± 31.65	161.02 ± 21.96	136.41 ± 13.88	**<0.001**^**†**^**:** *1–4: 0.001, 2–4: 0.002*
* LV-EDV-I/BSA (ml/m*^*2*^*)*	93.24 ± 12.80	85.77 ± 15.66	78.18 ± 10.06	67.48 ± 6.28	**<0.001**^**†**^**:** *1–4: <0.001, 2–4: 0.008*
* LV-EDV-I/H (ml/m)*	98.77 ± 15.49	96.55 ± 17.30	88.03 ± 10.90	77.64 ± 7.36	**0.001**^**†**^**:** *1–4: 0.001, 2–4: 0.005*
* LV-SV (ml)*	106.82 ± 20.49	108.45 ± 20.22	94.70 ± 18.34	82.40 ± 9.71	0.003*:*1–4: 0.006, 2–4: 0.004*
* LV-SV-I/BSA (ml/m*^*2*^*)*	55.31 ± 7.98	52.42 ± 9.82	45.96 ± 8.47	40.78 ± 4.68	**<0.001**^**†**^**:** *1–4: <0.001, 2–4: 0.007*
* LV-SV-I/H (ml/m)*	58.57 ± 9.59	59.05 ± 11.12	51.77 ± 9.58	46.91 ± 5.39	0.005*: *1–4: 0.012, 2–4: 0.010*
* LV-EF (%)*	59.39 ± 4.38	61.13 ± 3.42	58.50 ± 3.64	60.48 ± 4.90	0.368*
* LVM (g)*	100.19 ± 21.36	106.73 ± 26.89	98.30 ± 15.15	91.20 ± 16.34	0.335*
* LVM-I/BSA (g/m*^*2*^*)*	51.80 ± 8.56	51.45 ± 12.35	47.76 ± 7.44	44.81 ± 5.64	0.211*
* LVM-I/H (g/m)*	55.00 ± 10.67	58.14 ± 14.93	53.73 ± 7.75	51.75 ± 8.00	0.520*
* LV-MVR (g/ml)*	0.56 ± 0.10	0.60 ± 0.10	0.61 ± 0.05	0.67 ± 0.09	0.034*: *1–4: 0.024*
* RV-EDV (ml)*	203.54 ± 39.77	209.98 ± 40.60	189.77 ± 27.89	149.76 ± 17.27	<0.001*: *1–4: 0.001, 2–4: <0.001*
* RV-EDV-I/BSA (ml/m*^*2*^*)*	105.34 ± 15.34	101.33 ± 18.74	92.08 ± 12.93	74.05 ± 7.44	<0.001*: *1–4: <0.001, 2–4: <0.001*
* RV-EDV-I/H (ml/m)*	111.56 ± 18.14	114.29 ± 21.99	103.73 ± 14.32	85.22 ± 8.98	<0.001*: *1–4: 0.002, 2–4: <0.001*
* RV-EF (%)*	50.77 ± 4.08	51.18 ± 3.98	49.85 ± 5.00	50.85 ± 5.01	0.916*
* RV/LV-VR*	1.13 ± 0.07	1.18 ± 0.10	1.18 ± 0.12	1.10 ± 0.05	0.077^†^

*BSA* body surface area, *EDV* end-diastolic volume, *EF* ejection fraction, *H* height, *I* index, *LV* left ventricle, *LVM* left ventricular mass, *MVR* mass-to-volume ratio, *RV* right ventricle, *SD* standard deviation, *SV* stroke volume, *VR* volume ratio, *y* years. Statistics: *ANOVA with pairwise post-hoc parametric test, ^†^Kruskal-Wallis test with pairwise post-hoc non-parametric test. *P*≤0.05 defines statistical significance (bold)

RV/LV-VR was constant across all age groups in females and males, with young volunteers (age group 1) presenting almost similar RV/LV-VR parameters compared to volunteers in age group 4 (Tables 4, 5; Fig. 3). No significant correlation of RV/LV-VR with age for both sexes was found (Fig. 4).

LV-MVR increased significantly with rising age for both sexes (Tables 4, 5; Fig. 2), which was also indicated through a significant positive age-related correlation for LV-MVR (Spearman’s rank correlation coefficient: females +0.336, *p*=0.003, males +0.369, *p*=0.003, Fig. 4).

In males, LVM (and LVM indexed to BSA/H) did not differ significantly between the age groups but a trend towards an age-associated decline in LVM could be seen (Table 5; Fig. 2). Among females, LVM (and LVM indexed to BSA/H) decreased significantly between youngest (age group 1) and middle-aged groups (LVM and LVM-I/H age group 2, LVM-I/BSA age groups 2 and 3) but insignificantly between youngest (age group 1) and oldest (age group 4) subgroup (Table 4, Fig. 2).

### Intra- and interobserver-variability

**Fig. 5:**
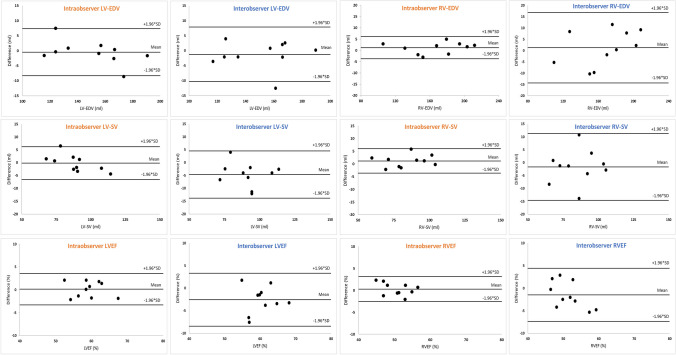
Results of intra (orange)- and interobserver (blue) analysis. The middle line represents the mean deviation, the upper line mean deviation + 1.96*SD, and the lower line mean deviation −1.96*SD. Abbreviations: LV-EDV, left ventricular end-diastolic volume; LVEF, left ventricular ejection fraction; LV-SV, left ventricular stroke volume; RV-EDV, right ventricular end-diastolic volume; RVEF, right ventricular ejection fraction; RV-SV, right ventricular stroke volume; SD, standard deviation

Bland-Altmann Plots for intra- and interobserver analysis are displayed in Figure 5. Mean deviations ± SD are provided in the supplementary results 2.

## Discussion

This study characterized adaptations in cardiac morphology and function during healthy aging in women and men using quantitative CMR indices and found that age was associated with several ventricular changes in both sexes even in younger life decades. First, there was evidence of equal ventricular remodeling in women and men with increasing age, as represented by an increase in concentricity (LV mass-to-volume ratio). Second, LV stroke volume index as a determinant of cardiac output decreased with rising age in both sexes but declined stronger for men than for women. Third, in both sexes, chamber volumes decreased with age with a similar decline in RV and LV so that the volume ratio between both chambers remained constant over the adult lifetime.

Overall, the major sex-specific differences in cardiac structure and function were found to be women having generally smaller ventricular dimensions and volumes, lower left ventricular mass as well as higher biventricular ejection fractions. These results confirm many studies that have established sex-specific reference ranges for quantitative CMR parameters [24, 28, 29]. Likewise, the results of the mentioned studies demonstrated that chamber dimensions decrease with rising age. This seems to be one of the fundamental age-related physiological cardiac adaptations in both sexes. Additionally, by using RV/LV-VR for quantitative assessment, this research revealed that the size of LV and RV declined equally with age in both sexes, represented by a preserved RV/LV-VR. Thus, age as a non-modifiable risk factor may not lead to an excessive workload on a particular single ventricle and a deviation of RV/LV-VR seems to indicate a specific underlying pathology. However, there is currently only limited knowledge about the clinical relevance of RV/LV-VR in CMR. Altmayer et al. demonstrated that the use of RV/LV-VR for diagnostic assessment increases the sensitivity for detecting RV dilatation in patients with pulmonary arterial hypertension [30]. Especially in congenital heart diseases, it was shown that RV/LV-VR seems to be a sex-neutral universal measure for the assessment of RV dilatation and pulmonary regurgitation as illustrated in patients with repaired tetralogy of Fallot [31]. However, the impact of age on the development of RV/LV-VR remains unexplained. Thus, this research, to the best of our knowledge, provides the first relevant CMR insights into the age-associated progression of RV/LV-VR, implicating that this parameter could serve as an age-independent diagnostic measure.

Another essential cardiac alteration was found by an age-associated increase in concentricity (LV-MVR) in both sexes. This ventricular adaptation cannot be explained by an increase in LVM with age, but by the stronger decline of LV end-diastolic volume in relation to LVM. This adjustment reflects rather a remodeling than a hypertrophy. These results confirm the findings of the CMR study by Cheng et al. [17], who investigated age-related ventricular alterations in the large MESA cohort. The study also found that LVM only decreased slightly in the elderly in both sexes, a trend that was similarly observed in this research (insignificant between youngest and oldest age groups), although there were slight differences in the population between MESA and the cohort of this study, which included younger healthy volunteers without traditional cardiac risk factors (e.g., arterial hypertension or hyperlipidemia). These findings were also supported in the longitudinal follow-up trial of the MESA cohort by Eng et al. [32], except for a long-term increase in LVM in men, which has not been reported before. Another healthy but sex-unspecific sample was studied by Kersten et al. [16], who found that LVM and indexed LVM decreased significantly with age while LV-MVR tended to increase in the elderly (insignificantly). Nevertheless, the age-related maintaining or decreasing LVM measured in CMR studies disagrees with echocardiographic trials that reported an age-associated increase in LVM [8, 9]. Representing the currently most important routine imaging modality in cardiology, echocardiography relies on different quantification methods which may influence the quantification of LVM. It was shown that LVM was larger when measured by echocardiography compared to CMR [33–35].

There were no clinically relevant sex-specific differences regarding the age-associated development of the above parameters (except for greater LV-MVR and LVM in males). In that regard, these results differ from previous studies that described a higher prevalence of concentric remodeling in females [10, 13, 36, 37]. This may be attributed to a different selection of participants, including the presence of cardiac diseases or risk factors compared to the healthy sample of this research.

Further, a sex-specific difference in the age-related progression of the LV stroke volume index as a determinant of cardiac output could be found. Although there were no significant age-associated changes in LV or RV ejection fractions in both sexes, the LV stroke volume index declined, namely stronger in men compared to women. Literature confirms an age-associated decline of LV stroke volume (index) [16, 17, 32]. Remarkably, the progression of LV-SVI differed slightly depending on whether it was indexed to BSA or H, even though both indices decreased with rising age. Indexed to BSA (weight-dependent), LV-SVI decreased faster compared to LV-SVI indexed to H, especially in males. Generally, the body characteristics of this cohort were comparable to other large populational-based studies [38, 39].

The cellular composition of the ventricular myocardium could provide a possible explanation for the higher biventricular systolic performance and the lower age-related decline in LV-SVI in women. Litviňuková et al. mentioned higher proportions of ventricular cardiomyocytes in women despite an overall lower total mass compared to men [40].

Given the sex-specific crossing point of the age-correlated LV-SVI/BSA trend curves in the years of early menopause onset, one could hypothesize that women entering menopause potentially undergo specific cardiac adaptations that are caused by changes in the sex-hormonal profile. Estrogen was generally associated with numerous cardioprotective mechanisms and higher postmenopausal estrogen levels were associated with a lower risk for cardiac diseases [5, 41–43]. Consequently, it is surprising that especially older women with normally decreasing estrogen levels demonstrate preserved and, in some cases, higher systolic LV performance. Further sex hormones can potentially contribute to postmenopausal cardiac changes: Subramanya et al. demonstrated that an androgenic hormone pattern (higher free testosterone, lower sex hormone binding globulin levels) was connected to an increase in LVM and concentricity in postmenopausal women [44]. Future research should aim to further investigate the impact of sex-hormonal changes upon pre- and post-menopausal women regarding the cardiac morphology as well as the ventricular function. Along with the investigation of biological characteristics, the sociocultural background and social identities of patients (gender rather than sex) may also be considered in further research. The scientific and clinical assessment of sociocultural environmental factors such as gender-specific health behavior, lifestyle, physical activity, and nutrition could improve the patient care and provide relevant information about sex- and gender-specific differences in the expression and development of cardiac diseases.

Interestingly, it was shown that a regression of atrial size occurs during aging in correlation with the decrease in ventricular size observed in healthy volunteers [45]. Further, associations with age-related changes in myocardial tissue composition are known as T1-mapping times appear to increase with age, while T2-mapping times tend to decrease [46]. Women additionally demonstrated significantly higher T1-mapping times than men [46, 47]. But, published data in this regard are still conflicting. One could assume that this is related to different study cohorts and techniques. An integrated analysis of atrial and ventricular remodeling including myocardial tissue characterization as well as hemodynamic assessment could be helpful for a deeper understanding of physiological changes in the sex-specific aging heart.

### Study limitations

This analysis was based on a retrospective and single-center design. As the identification and inclusion of healthy participants is more challenging with rising age, the participants in each age group could not be equally balanced. A comprehensive analysis of atria, parametric mapping–based myocardial tissue analysis, and phase contrast technique–based flow assessment were not possible as the data were not available in all volunteers. For ethical requirements, a contrast agent could not be applied to healthy volunteers.

## Conclusions

By using CMR, this study found several physiological structural and functional heart adaptations during healthy aging. Both women and men showed comparable age-associated ventricular adjustments, as illustrated in the similar increase in concentricity as well as the general decrease of ventricular dimensions. Nevertheless, the female heart cannot just be considered a smaller version of the male’s heart. This study found higher biventricular systolic function and a smaller age–related decline in systolic LV performance in women. To extend knowledge about underlying causes of sex-specific cardiovascular adaptations over a lifetime, additional research will be required.

## Supplementary Information

Below is the link to the electronic supplementary material.

Supplementary methods 1 (PDF 292 KB)

Supplementary methods 2 (PDF 85 KB)

Supplementary results 1 (PDF 420 KB)

Supplementary results 2 (PDF 109 KB)

## Data Availability

Data are available from the corresponding author upon reasonable request.
