# Supplementary material for: Sex-specific structural and functional cardiac remodeling during healthy aging assessed by cardiovascular magnetic resonance
Source: Clin Res Cardiol. 2024 Mar 11;114(12):1619–30. doi: 10.1007/s00392-024-02430-5 (PMC12708725; doi:10.1007/s00392-024-02430-5)
Supplement: Supplementary file 1 — Supplementary methods 1 (PDF 292 KB) [file 392_2024_2430_MOESM1_ESM.pdf]

Supplementary methods 1

Article title:
Sex-specific structural and functional cardiac remodeling during healthy aging assessed by cardiovascular magnetic resonance.

Journal:
Clinical Research in Cardiology

Authors:
Leonhard Grassow[a,b,c], Jan Gröschel[a,b,c,d], Hadil Saad[a,b,e], Leo Dyke Krüger[a,b,c], Johanna Kuhnt[a,b,c], Maximilian Müller[a,b,c], Thomas Hadler[a,b,c], Edyta Blaszczyk[a,b,c], Jeanette Schulz-Menger[a,b,c,e*]

Author affiliations:
[a]Charité – Universitätsmedizin Berlin, corporate member of Freie Universität Berlin and Humboldt-Universität zu Berlin, ECRC Experimental and Clinical Research Center, Lindenberger Weg 80, 13125 Berlin, Germany

[b]Working Group on Cardiovascular Magnetic Resonance, Experimental and Clinical Research Center, a joint cooperation between Charité Medical Faculty and the Max-Delbrück Center for Molecular Medicine, Berlin, Germany

[c]DZHK (German Centre for Cardiovascular Research), partner site Berlin, Germany

[d]Deutsches Herzzentrum der Charité – Department of Cardiology, Angiology and Intensive Care Medicine, Charitéplatz 1, 10117 Berlin, Germany

[e]HELIOS Hospital Berlin-Buch, Department of Cardiology and Nephrology, Schwanebecker Chaussee 50, 13125 Berlin, Germany

*Corresponding author:
Univ.-Prof. Dr. med. Jeanette Schulz-Menger
E-Mail: jeanette.schulz-menger@charite.de

Supplementary methods 1 - Study enrollment:

203 caucasian volunteers were retrospectively screened for this study. All of them were enrolled as healthy controls in previous prospective studies and underwent cardiovascular magnetic resonance (CMR) imaging. Participants should have been at least 18 years or older and there was no upper age limit. The initial recruitment followed a standard operating procedure (SOP): volunteers were interviewed regarding their medical history and underwent a physical examination prior to their inclusion. All information were documented in case report files (CRFs). Exclusion criteria were defined as follows: Cardiovascular diseases or symptoms:

angina pectoris, dyspnea, coronary or peripheral artery disease, heart failure (HFrEF, HFmrEF, HFpEF), valve disease (any degree of severity), arrhythmias, arterial hypertension, treated dyslipidemia, structural heart disease and major cardiovascular events such as myocardial infarction, stroke or cardiac surgery. Exclusion criteria were further extended to conditions beyond the cardiovascular system and considered chronic inflammatory skin diseases (e.g. psoriasis, atopic dermatitis), autoimmune diseases (e.g. sjogren's syndrome, thyroid disease), pulmonary (e.g. bronchial asthma), neurological (e.g. migraine) and gastroenterological disorders (e.g. reflux esophagitis) as well as other inflammatory conditions (e.g. non-specific upper respiratory tract infections) that were temporally related to the CMR examination. Participants with missing CMR image datasets (missing basal layering, incompletely displayed left- or right ventricle in short-axis stack, completely missing short-axis view) or poor image quality (major artefacts such as image blurring, mis-triggering, or wrap-around artefacts, leading to inability of analysis) were also excluded from the cohort. Supplementary Figure 1 (below) displays the study enrollment.

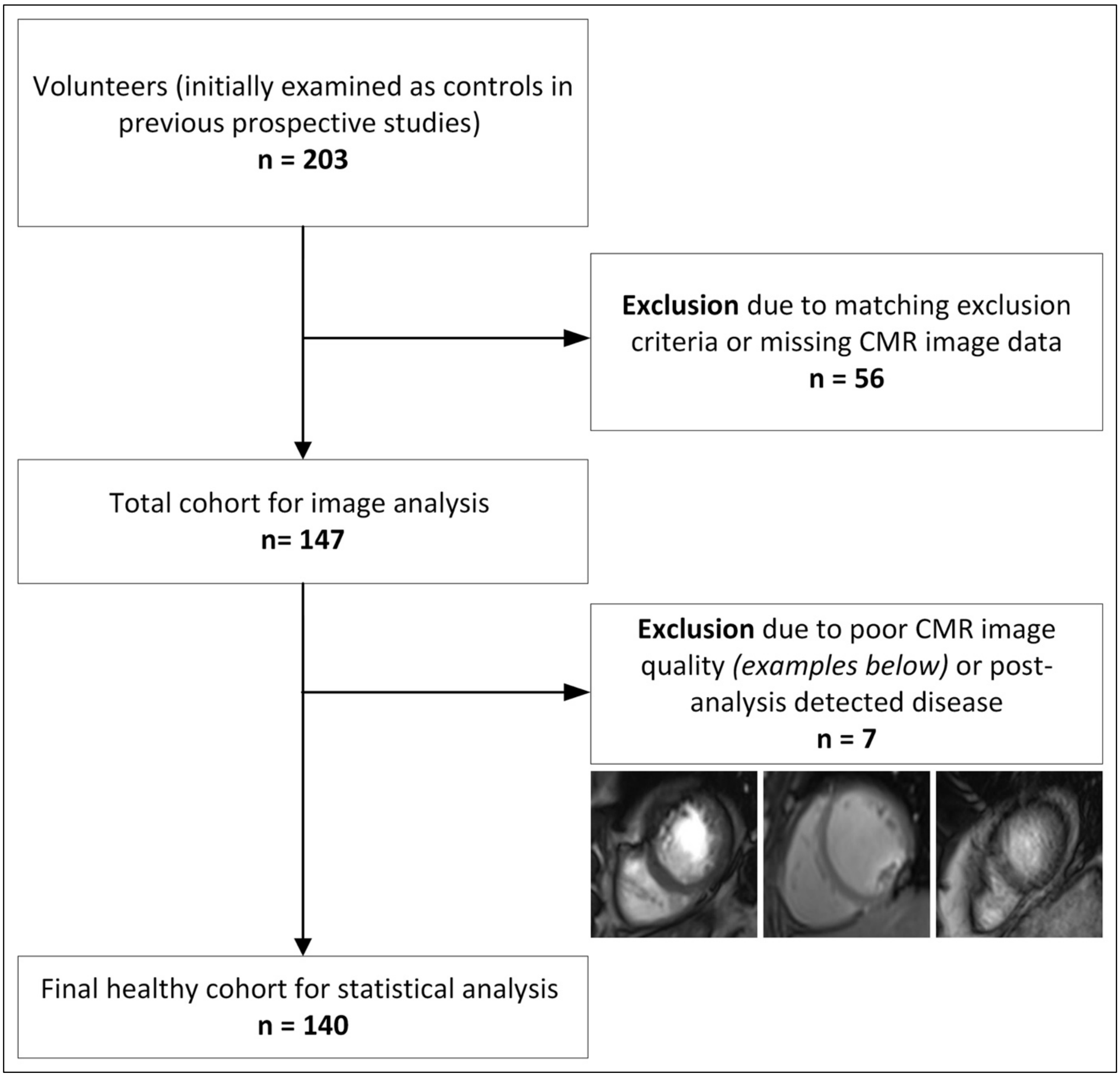

Supplementary Fig. 1 Study enrollment. Abbreviation: CMR: Cardiovascular magnetic resonance.
