# Supplementary material for: Sex-specific structural and functional cardiac remodeling during healthy aging assessed by cardiovascular magnetic resonance
Source: Clin Res Cardiol. 2024 Mar 11;114(12):1619–30. doi: 10.1007/s00392-024-02430-5 (PMC12708725; doi:10.1007/s00392-024-02430-5)
Supplement: Supplementary file 2 — Supplementary methods 2 (PDF 85 KB) [file 392_2024_2430_MOESM2_ESM.pdf]

Supplementary methods 2

Article title:
Sex-specific structural and functional cardiac remodeling during healthy aging assessed by cardiovascular magnetic resonance.

Journal:
Clinical Research in Cardiology

Authors:
Leonhard Grassow[a,b,c], Jan Gröschel[a,b,c,d], Hadil Saad[a,b,e], Leo Dyke Krüger[a,b,c], Johanna Kuhnt[a,b,c], Maximilian Müller[a,b,c], Thomas Hadler[a,b,c], Edyta Blaszczyk[a,b,c], Jeanette Schulz-Menger[a,b,c,e*]

Author affiliations:
[a]Charité – Universitätsmedizin Berlin, corporate member of Freie Universität Berlin and Humboldt-Universität zu Berlin, ECRC Experimental and Clinical Research Center, Lindenberger Weg 80, 13125 Berlin, Germany

[b]Working Group on Cardiovascular Magnetic Resonance, Experimental and Clinical Research Center, a joint cooperation between Charité Medical Faculty and the Max-Delbrück Center for Molecular Medicine, Berlin, Germany

[c]DZHK (German Centre for Cardiovascular Research), partner site Berlin, Germany

[d]Deutsches Herzzentrum der Charité – Department of Cardiology, Angiology and Intensive Care Medicine, Charitéplatz 1, 10117 Berlin, Germany

[e]HELIOS Hospital Berlin-Buch, Department of Cardiology and Nephrology, Schwanebecker Chaussee 50, 13125 Berlin, Germany

*Corresponding author:
Univ.-Prof. Dr. med. Jeanette Schulz-Menger
E-Mail: jeanette.schulz-menger@charite.de

Abbreviations:

CMR: Cardiovascular magnetic resonance

bSSFP: balanced steady-state free precession

LV: Left ventricle

RV: Right ventricle

Supplementary methods 2 – Technical CMR sequence details:

The analysis was performed using balanced steady-state free precession (bSSFP) CMR cine imaging acquisitions in a short-axis stack covering the whole LV and RV. Technical CMR sequence details varied as follows:

At 1.5 Tesla scanners (Avanto and AvantoFit, Siemens Healthnieers, Erlangen, Germany): repetition time: 34.7 - 62 ms, echo time: 1.14 - 1.48 ms, flip angle: 70 - 80 degrees, fields of view: 255 - 308.75 x 340 - 380 mm^2, matrix: 144 - 216 x 192 - 256, slice thickness and gap: 7 / 0 mm and 7 / 3 mm, 30 cardiac phases.

At 3 Tesla scanners (SkyraFit and Verio, Siemens Healthineers, Erlangen, Germany): repetition time: 3.1 - 45.78 ms, echo time: 1.3 - 1.43 ms, flip angle: 45 - 54 degrees, fields of view: 255 - 301.15 x 340 - 360 mm^2, matrix: 156 - 174 x 192 - 208, slice thickness and gap: 7 / 0 mm, 6 / 0 mm and 7 / 3 mm, 30 cardiac phases.
