# Supplementary material for: Sex-specific structural and functional cardiac remodeling during healthy aging assessed by cardiovascular magnetic resonance
Source: Clin Res Cardiol. 2024 Mar 11;114(12):1619–30. doi: 10.1007/s00392-024-02430-5 (PMC12708725; doi:10.1007/s00392-024-02430-5)
Supplement: Supplementary file 3 — Supplementary results 1 (PDF 420 KB) [file 392_2024_2430_MOESM3_ESM.pdf]

Supplementary results 1

Article title:
Sex-specific structural and functional cardiac remodeling during healthy aging assessed by cardiovascular magnetic resonance.

Journal:
Clinical Research in Cardiology

Authors:
Leonhard Grassow[a,b,c], Jan Gröschel[a,b,c,d], Hadil Saad[a,b,e], Leo Dyke Krüger[a,b,c], Johanna Kuhnt[a,b,c], Maximilian Müller[a,b,c], Thomas Hadler[a,b,c], Edyta Blaszczyk[a,b,c], Jeanette Schulz-Menger[a,b,c,e*]

Author affiliations:
[a]Charité – Universitätsmedizin Berlin, corporate member of Freie Universität Berlin and Humboldt-Universität zu Berlin, ECRC Experimental and Clinical Research Center, Lindenberger Weg 80, 13125 Berlin, Germany

[b]Working Group on Cardiovascular Magnetic Resonance, Experimental and Clinical Research Center, a joint cooperation between Charité Medical Faculty and the Max-Delbrück Center for Molecular Medicine, Berlin, Germany

[c]DZHK (German Centre for Cardiovascular Research), partner site Berlin, Germany

[d]Deutsches Herzzentrum der Charité – Department of Cardiology, Angiology and Intensive Care Medicine, Charitéplatz 1, 10117 Berlin, Germany

[e]HELIOS Hospital Berlin-Buch, Department of Cardiology and Nephrology, Schwanebecker Chaussee 50, 13125 Berlin, Germany

*Corresponding author:
Univ.-Prof. Dr. med. Jeanette Schulz-Menger
E-Mail: jeanette.schulz-menger@charite.de

Supplementary results 1 – Additional age-correlated left ventricular parameters:

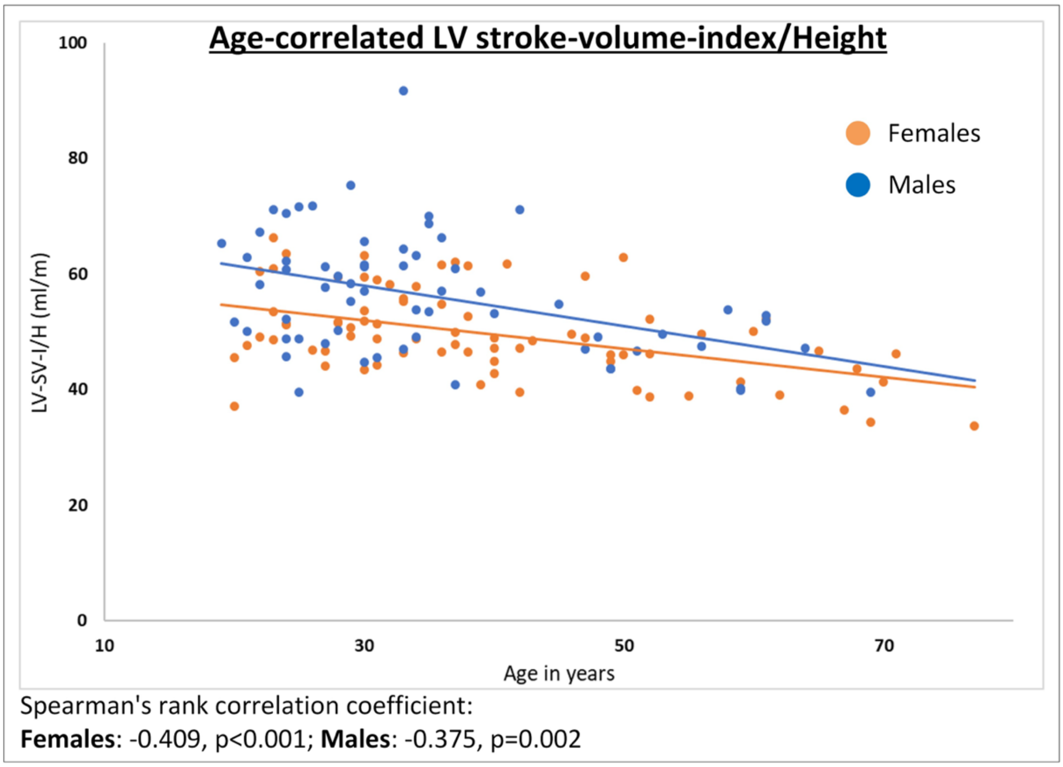

Supplementary Fig. 2 Age-correlated development of LV stroke-volume-index/Height in women and men. Female graph: orange, male graph: blue. Abbreviations: H: Height, I: Index, LV: Left ventricle, SV: Stroke volume.

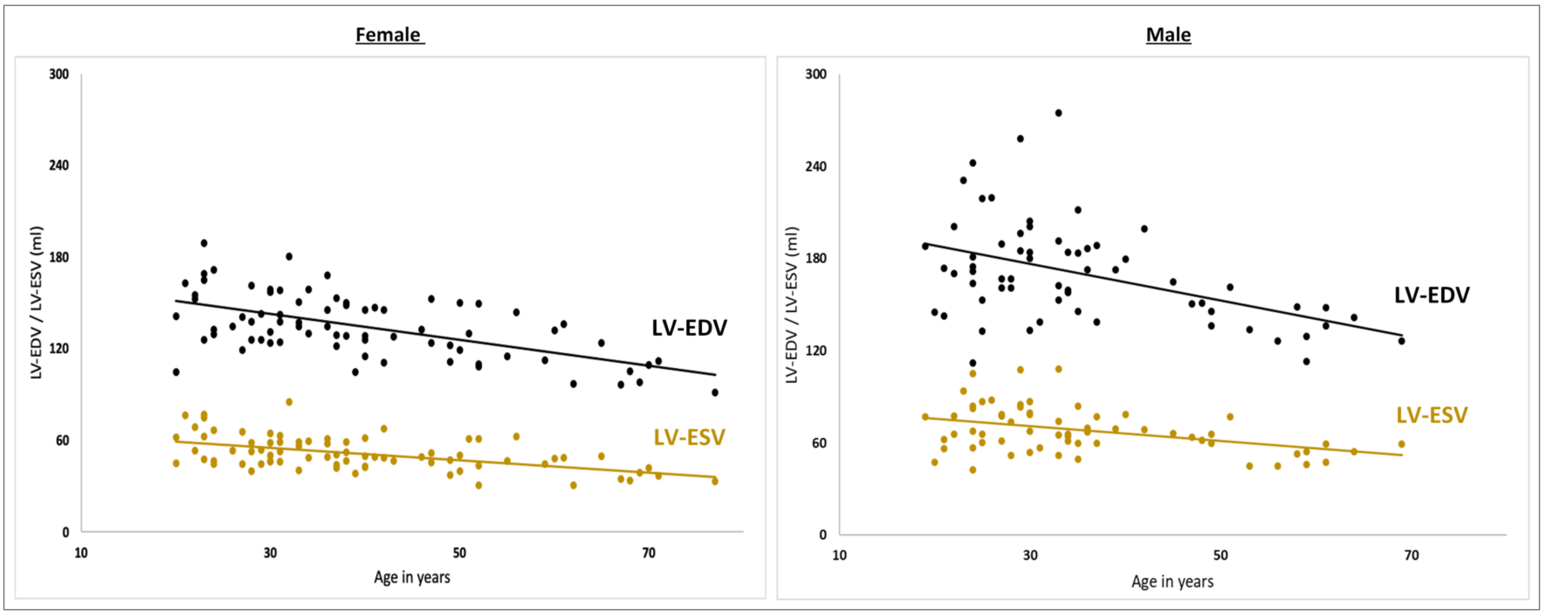

Supplementary Fig. 3 Age-related development of LV end-systolic-volume (ocher) and end-diastolic-volume (black) in women (left) and men (right). Abbreviations: LV: Left ventricle, EDV: End-diastolic-volume, ESV: End-systolic-volume.
