# Supplementary material for: Sex-specific structural and functional cardiac remodeling during healthy aging assessed by cardiovascular magnetic resonance
Source: Clin Res Cardiol. 2024 Mar 11;114(12):1619–30. doi: 10.1007/s00392-024-02430-5 (PMC12708725; doi:10.1007/s00392-024-02430-5)
Supplement: Supplementary file 4 — Supplementary results 2 (PDF 109 KB) [file 392_2024_2430_MOESM4_ESM.pdf]

Supplementary results 2

Article title:
Sex-specific structural and functional cardiac remodeling during healthy aging assessed by cardiovascular magnetic resonance.

Journal:
Clinical Research in Cardiology

Authors:
Leonhard Grassow[a,b,c], Jan Gröschel[a,b,c,d], Hadil Saad[a,b,e], Leo Dyke Krüger[a,b,c], Johanna Kuhnt[a,b,c], Maximilian Müller[a,b,c], Thomas Hadler[a,b,c], Edyta Blaszczyk[a,b,c], Jeanette Schulz-Menger[a,b,c,e*]

Author affiliations:
[a]Charité – Universitätsmedizin Berlin, corporate member of Freie Universität Berlin and Humboldt-Universität zu Berlin, ECRC Experimental and Clinical Research Center, Lindenberger Weg 80, 13125 Berlin, Germany

[b]Working Group on Cardiovascular Magnetic Resonance, Experimental and Clinical Research Center, a joint cooperation between Charité Medical Faculty and the Max-Delbrück Center for Molecular Medicine, Berlin, Germany

[c]DZHK (German Centre for Cardiovascular Research), partner site Berlin, Germany

[d]Deutsches Herzzentrum der Charité – Department of Cardiology, Angiology and Intensive Care Medicine, Chariteplatz 1, 10117 Berlin, Germany

[e]HELIOS Hospital Berlin-Buch, Department of Cardiology and Nephrology, Schwanebecker Chaussee 50, 13125 Berlin, Germany

*Corresponding author:
Univ.-Prof. Dr. med. Jeanette Schulz-Menger
E-Mail: jeanette.schulz-menger@charite.de

Abbreviations:

LV-EDV: Left ventricular end-diastolic-volume

LV-EF: Left ventricular ejection fraction

LV-SV: Left ventricular stroke volume

RV-EDV: Right ventricular end-diastolic-volume

RV-EF: Right ventricular ejection fraction

RV-SV: Right ventricular stroke volume

Supplementary results 2 – Intra- and interobserver variability:

The mean deviations ± standard deviations (SD) for intra- and interobserver variability were as follows:

Intraobserver analysis:

LV-EDV (ml): -0.48 ± 4.00; RV-EDV (ml): 1.18 ± 2.52; LVEF (%): 0.14 ± 1.75; RVEF (%): 0.29 ± 1.44; LV-SV (ml): -0.19 ± 3.26; RV-SV (ml): 1.18 ± 2.46.

Interobserver analysis:

LV-EDV (ml): -1.23 ± 4.64; RV-EDV (ml): 1.28 ± 7.96; LVEF (%): -2.55 ± 3.00; RVEF (%): -1.46 ± 3.00; LV-SV (ml): -4.67 ± 4.68; RV-SV (ml): -1.66 ± 6.61.
